# A Market of Lived Experience—User Involvement and the Commodification of Personal Experiences of Mental Illness

**DOI:** 10.3390/ijerph20146427

**Published:** 2023-07-22

**Authors:** Erik Eriksson

**Affiliations:** Faculty of Social Sciences, School of Social Work, Lund University, 22100 Lund, Sweden; erik.eriksson@soch.lu.se

**Keywords:** service user involvement, commodification, experts by experience, mental health, narrative

## Abstract

Working actively to engage service users in participatory practices is both a policy expectation and a moral imperative for mental health social workers in contemporary Western mental health care. Recent research suggests that such practices of service user involvement are becoming increasingly individualised and driven by market logic. Based on an ethnographic study within a Swedish public psychiatric organisation, this article applies the concept of commodification to examine this trend. By showing how the practice of user involvement takes the form of a market where personal narratives and experiences of mental health problems are bought and sold as commodities, the analysis illuminates how market logic permeates the everyday practice of user involvement. One consequence of this commodification is that user organisations, as well as individual service users, are restricted in their role as independent actors pursuing their own agenda, and instead increasingly act on behalf of the public and as providers of personal experiences. While it is vital that service user perspectives are heard and recognised within mental health services, mental health social workers need to be aware of the risks of commodifying lived experience. When attention is directed to individual experiences and narratives, there is a risk that opportunities to advocate on behalf of the user collective as a whole and speak from a more principled and socio-political standpoint are lost. In addition, the commodification of personal experience tends to rationalise and privilege user narratives that conform to the dominant institutional logic of the mental health organisation, while excluding more uncomfortable and challenging voices, thereby undermining the ability of service users to raise critical issues that do not align with the interests of the mental health organisation.

## 1. Introduction

In 2019, an earlier version of this text was published in Swedish by the Swedish Sociology Association (SSA) in the journal *Sociologisk forskning* (‘Sociological research’ 2019, 56, 85–109). The article has been reworked, translated into English, and republished with the full permission of the SSA. This is the first and only version of the article to be published in English.

In addition to being a scholar of social work—entering my thirteenth year in academia, where I have spent most of my time researching user perspectives and participation within public welfare services—I am also a social worker by profession. As a young, aspiring social worker, I became interested in mental health issues, working both in a state psychiatric hospital and later in the Fountain House movement. During these years, like many mental health social workers before me, I realised the importance of listening to and drawing on the experiences of people who use the services [1]. Therefore, this article will not reflect my personal story, nor the narratives of mental health social workers per se, but the experiences of mental health service users and survivors. A crucial, but perhaps somewhat overlooked, issue for mental health social workers and other mental health professionals is how to incorporate user perspectives into mental health services. Through this analysis, I will provide a critical account of how the lived experience of mental illness is increasingly, and perhaps unreflectively, being treated as a commodity within mental health services, and what the consequences of this might be. But let us begin the exploration in the popular culture of society at large—the therapeutic society, as it might be called [2]—where representations of mental illness are equally gaining attention.

Therése Lindgren runs one of Sweden’s most popular YouTube channels, with around ten million views a month. She states on her website that she has ‘the best job in the world’. Recurring themes in Therese’s texts and videos are cooking, beauty, and mental health issues. She is also the author of the book *Sometimes I don’t feel well* [3], which describes her anxiety syndrome and sold over 100,000 copies in its first month. Hence, part of Therese’s work—part of what she sells—are accounts of her personal experience of mental illness. In Sweden alone, there are many examples of people who make a living, in whole or in part, from recounting their experiences of mental health problems and psychiatric treatment, including Michaela Aspelin [4], Ann Heberlein [5], and Christian Dahlström [6], to name a few. Several of these authors, as well as many others, also give talks about their experiences. (Prominent international examples include, among numerous others, Claire Eastham [7], Rose Bretécher Cartwright [8], and Bryony Gordon [9].) There seems to be a societal interest—a demand, to put it in economic terms—for personal testimonies about mental illness and psychiatric care. In what follows, I will explore what happens when this demand for personal testimonies extends into the public mental health system and its implementation of service user involvement. (This empirical study examines service user involvement at an organisational level. Thus, this article deals with involvement practices that enable the target group to participate in policymaking, strategy, and development at a level above the individual case.) As individual lived experience becomes increasingly popular as a source of knowledge within user involvement practices, it is becoming common for patients and former patients to be offered financial compensation for sharing their stories. What is occurring can be understood in terms of commodification, where experiences of mental illness become an asset and a commodity that can be bought and sold in a market.

Many people who experience mental ill-health live in constrained economic circumstances [10], so the opportunity to monetise their experiences can provide much-needed income. The dissemination of personal narratives is also highlighted as a way to reduce the stigma of mental illness and improve mental health services and treatment [1,11]. However, previous research suggests that when personal user narratives are incorporated into public psychiatry, they risk losing their critical dimension and potential for change [12,13,14,15]. Furthermore, such incorporation may also have a detrimental effect on the bearer of the personal experience [15,16]. Therefore, it is crucial to ask what implications the commodification of lived experience may have for those whose experiences become commodified and examine how such commodification may affect the prospects of the service user collective to contribute to what they identify as desirable outcomes [17].

In Sweden, as in most Western welfare states, user involvement has been on the policy agenda for more than 30 years [18,19,20], and the phenomenon has changed over time. For example, contemporary user involvement is increasingly individualised [21] and influenced by market logic [22]. Using the commodification of lived experience as an example, this article explores how these trends are expressed in practice. Based on an ethnographic study of user involvement within a public organisation for specialised mental health care, the article explores two interrelated questions: (1) how lived experiences of mental illness and psychiatric treatment are constituted as commodities and (2) how the market for these experiences manifests itself. This analysis concludes with a reflection on the consequences of these commodification tendencies for service users and their ability to exercise influence and a discussion on the implications for mental health social workers (and other professionals) who aim to create meaningful participatory processes. Before moving on to the analysis, however, I would like to comment briefly on three central aspects of the text: service user involvement (the empirical case), commodification (the main theoretical concept), and the research methods.

## 2. Service User Involvement and Its Development

### 2.1. An Ambiguous Concept

Briefly, user involvement can be defined as the attempt to allow the target group of a human service organisation to influence the formation and implementation of the organisation’s activities. However, such a simple definition ignores the complexity of the phenomenon. User involvement is an ambiguous phenomenon, defined at any given time and place at the intersection of different expectations, ideals, and purposes.

Two basic logics of user involvement are often identified: a democratising ambition and an organisational instrumental ambition [18,19]. The former can be linked to the rise of the user movement which, inspired by the trade union, human rights, and anti-psychiatry movements of the 1960s and 1970s, began to demand influence over welfare state arrangements [23]. Here, the idea of organised user involvement was born out of demands for change in a public administration that was perceived as paternalistic and stigmatising. The aim was to build more democratic human service organisations where the power of professionals was shared, and the user collective had the opportunity to influence services. Croft and Beresford [18] have termed this the ‘democratic approach’.

In the 1970s, the user movement was strong—in Sweden as in the UK—and with the active support of professionals in academia, psychiatry, and the media, it was an important voice in the public debate [23,24]. Over time, the state adapted, and user involvement practices gradually became institutionalised within the public welfare system. This gave rise to the second ideal starting point for user involvement, which Croft and Beresford [18] termed the ‘managerialist/consumerist approach’. This logic focuses on organisational functionality and is associated with the introduction of new public management in the public sector. Here, user involvement is formulated to evaluate and create more efficient services that are better adapted to the needs of service users—the consumers [25].

In practice, the ambitions associated with these two ideal logics coexist and intertwine. In addition, a new starting point for user involvement has gained ground, the so-called ‘co-production perspective’, in which the user group is formulated as a resource that should be involved—through various forms of cooperation—as co-creators and providers of welfare services [26,27,28].

### 2.2. The Wider Context

User involvement exists within a wider context of political and social trends that influence its formulation. For example, modern society is characterised by a pronounced individualisation [29,30], where the individual’s identity, autonomy, rights, and self-realisation occupy a historically unprecedented place in the perception of life and society. This individualisation is linked to the expansion of liberal ideology [30], which has also accentuated work as the way to become a complete citizen [31,32] and led to the marketization of previously public sectors [33]. In the welfare sector, the neoliberal turn has led to austerity policies [34,35], workfare policies [36,37], and the privatisation and creation of quasi-markets in health and social care [38,39].

The formulation of user involvement has changed with the spirit of the times, from a left-wing intellectual starting point in the sixties and seventies to a more neoliberal contemporary orientation. The Swedish political formulation of user involvement, which in the early 1990s was dominated by ideas about democracy and collective influence, has become increasingly individualised and linked to issues of organisational efficiency [21,40]. Moreover, contemporary political interest in user involvement has been linked to the ambition to create citizens responsible for their own welfare [41]. Linked to this development, the state has sought to transfer some of the responsibility for delivering welfare services to the voluntary sector [26], an ambition that has met with resistance from civil society organisations in Sweden [42].

## 3. Commodification

The concept of commodification derives from Marx’s early works (see [43]) and has been developed throughout Marxist theory (see, for example, [44,45]). Originally, commodification described what happened after the Industrial Revolution, when the capitalist market economy emerged as the dominant logic for organising wealth and livelihoods in society. Commodification meant that the material conditions of life—land, housing, means of production, money, supplies, and labour—became commodities that could be bought and sold in a market. In particular, the commodification of individual labour is seen as a prerequisite for the market economy and class society [43,44]. Moreover, Marx argued that this commodification drained individuals of their identity and basic humanity: it alienated people from themselves and from other people [43].

The fact that the livelihood of individuals (workers) became dependent on their ability to sell their labour to those who owned the means of production is central to Marxist theory, and the Marxist (and later socialist) political project aimed to abolish (or limit) individuals’ dependence on the market to secure their wealth. The introduction of social rights [46] and the development of welfare states [47] became ways of limiting this dependence and creating decommodification [47] (pp. 21–22). However, with the expansion of the modern market system, welfare arrangements have been curtailed (and themselves commodified), leading contemporary theorists to speak of recommodification, where individuals’ wealth is again more dependent on their ability to sell their labour [36,37].

In contemporary Western society, it is not only the material aspects of life that are commodified. The social and cultural dimensions of life are also increasingly permeated by market logic, leading Harvey [30] to speak of the ‘commodification of everything’ (p. 165). Identity [48], sexuality [49], religion [50], emotions [51], art [52], and cultural heritage [53] are examples of phenomena considered to be commodified, as is education [54] and academic research [55].

The implications of commodification vary with the phenomenon studied. Moreover, the fact that a particular field or phenomenon is commodified does not mean that this is the only significant aspect that characterises the phenomenon or that it can only be understood in terms of commodification. With regard to my object of study—the lived experience of mental illness and the articulation of such narratives—it can be understood, for example, as an act of resistance [14], as a way of creating learning [11,17], as a tool for recovery [56], or as a way of communicating opinions and asserting influence [57]. The implications of a personal narrative vary depending on the context. All of the above ways of interpreting narratives of mental illness are possible and are not precluded by the fact that the same narratives are simultaneously affected by—or an expression of—processes of commodification.

However, simply stating that a field is commodified is of limited analytical value. More interesting is to examine how such commodification manifests and operates. The aim of this article is, therefore, to clarify how the field of user involvement takes the form of a market in which the lived experience of mental illness is constituted as a commodity and analyse the plausible consequences of this.

### The Commodification of Welfare and User Involvement

Since the 1980s, the welfare sector has also been commodified [39,58]. Individuals’ welfare and health have become a commodity that private actors compete for in a welfare market [38,59] or a service that those who can afford it can pay for [60]. In the context of welfare, it has been noted that social movements and civil society actors have also been subject to commodification [61], where the latter are increasingly expected to devote themselves to selling services to the public [42,62]. Mental health and psychiatric treatment have also been seen as commodified [63,64], not least through the influence of the pharmaceutical industry [65].

Similarly, user involvement practices are also influenced by market logic [22], and the development demonstrated, for example, by Askheim and colleagues [27] and Alm Andreassen [28]—where the issue of involvement is formulated in terms of ‘co-production’—can be interpreted as a manifestation of commodification. Here, user involvement is implemented by service users and user organisations selling their expertise and skills to contribute to the ‘production’ of services. Furthermore, Lupton [66] has shown how service users’ opinions were commodified when users of social services reported their opinions on a web application; their responses were later resold for commercial purposes. Specifically in relation to service user narratives, Jijan Voronka, who identifies as a psychiatric survivor with experience of being employed to retell her personal story, has explored how narratives of mental illness become a product that is consumed in both mental health care [15] and social work education [17]. From a critical user perspective, she examines how this commodification has affected the content of narratives and the ability of user groups to pursue their goals, arguing in her doctoral thesis [15] that this process results in service users losing control of their own narratives as they are shaped to fit the institutional needs of organisations. This runs the risk of reducing the critical and transformative potential of the narratives and instead contributing to the re-production of dominant and mainstream perceptions of mental illness and mental health care (ibid). My article builds on Voronka’s research and extends our knowledge of the process by which experiences are commodified and how the market of lived experience manifests itself.

## 4. Material and Methods

This article is based on an ethnographic study of how user involvement at the organisational level has been realised within a regional (county-based) organisation for specialised mental health care (see [40]), referred to in the text as the Public Psychiatry Organisation or PPO. The organisation employs approximately 3000 people—with care assistants, nurses, social workers, psychologists, and psychiatrists as the most common occupations—and manages care units throughout the county, providing outpatient and inpatient care across the mental health spectrum for both adults and minors.

### 4.1. Data Collection and Data Analysis

Ethnographic studies aim to get as close as possible to the object of study. Participant observation is, therefore, a key method as it allows the researcher to study a practice as it happens [67]. The starting point of the project was to investigate how user involvement is constructed through practice, and the activities observed were those defined by the PPO as working with (organisational level) user involvement. Over the course of one year in 2011 and 2012, observations were made on 37 separate occasions, totalling 125 h, corresponding to approximately 600 A4 pages of field notes. At each observation, I informed those present about the study and asked for their consent. Where possible, I also informed participants of the study in advance, either verbally, by email, or by letter.

As emphasised by Maravasti [68], analysis and interpretation are ongoing throughout the research project, but once the fieldwork was completed and the field notes were written up, I undertook a structured thematic analysis using a common computer software package designed for qualitative analysis.. Ethnographic studies typically adopt an approach in which the empirical material (rather than hypotheses or theoretical frameworks) is used as a starting point for understanding the field [67]. Thus, the aim of the original analysis was to investigate how user involvement was constructed through organisational practice. In this respect, a number of ways of working with user involvement emerged, and through the analysis, I have grouped these into five categories: ‘Development work’ (participation in operational changes within the PPO), ‘Dialogue activities’ (forum for dialogue and consultation between the PPO and user representatives, such as user councils), ‘Opinion gathering’ (surveys and evaluations to capture user perceptions), ‘Co-production’ (participation in the day-to-day work of the organisation, for example by leading self-help groups or being employed as a staff member), and ‘Educational activities’ (participation in the training and competence development of the organisation’s staff). (A more comprehensive summary of the different ways of working with user involvement can be found in the English summary of my doctoral thesis [40] on p. 318.) Even though it was not assumed beforehand, a sub-theme that eventually emerged during the analysis had to do with market logic and commercialisation. Undoubtedly, many things were happening at the same time when user involvement was put into practice, and not all activities were dominated by such logic. In some activities, such as user councils and other dialogue activities, democratic logic was the dominant one. To some extent, however, traces of market logic and commercialisation appeared in all forms of involvement, particularly in activities where service users were employed to carry out tasks or participate in staff training programmes. This article highlights those parts of the empirical material that relate to the original sub-theme of ‘market logics and commercialisation’, which has subsequently been theorised in terms of commodification. In the excerpt from the field notes presented, all personal names, places, and other characteristics have been changed. The field notes are reconstructions of what was said and what happened, and quotation marks indicate exact wording.

### 4.2. The Field, Members of the Field, and Myself

The activities observed were usually meetings, lasting between two and three hours, but in some cases longer, such as all-day training sessions or conferences. Cross-administration work, such as the central user council, was often carried out in the PPO’s headquarters; a large 1960s brick building in one of the county’s larger cities, in cramped conference rooms with low ceilings. Perhaps for this reason, larger events were often held in hotels or conference centres. I also travelled to several other towns in the county to observe activities in local care units. These premises were rather homogeneous, with a ‘hospital environment’ feel; white walls, wide corridors with plastic floors, and the smell of disinfectants and medicines. Rarely were the premises or furniture new. We usually met in meeting, group, and conference rooms to which normally only staff had access.

The staff I usually encountered were nurses, carers, and social workers. Other common professions were psychologists and doctors, but they rarely took part in the activities and when they did, they tended to be in managerial positions. The central management of the PPO employed a ‘user involvement coordinator’ who became one of my main gatekeepers, and I observed many involvement activities that he arranged. In addition to this coordinator, each care unit had a local coordinator, and some of these also became some of my closest local contacts.

In large-scale activities, such as open space meetings, a whole population of service users (e.g., all the ‘patients’ of a particular PPO care unit) could be invited to participate, but more often a limited number of users were involved. In some of these activities, users acted as official representatives of a user organisation, and in others, they were involved on the basis of their individual experience as users. Sometimes this distinction was not clear, as users who were not representing a user organisation in a particular context could still be members of a user organisation. User representatives were of all ages and genders, but noticeably few were from migrant backgrounds. Several user organisations were closely involved in the PPO’s involvement activities, the two largest being the National Association for Social and Mental Health (RSMH) and the National Association for Rights, Liberation, Health, and Equal Treatment (RFHL).

A researcher cannot capture all aspects of a field or claim that the descriptions correspond to the actors’ understanding of reality. Therefore, as in social work practice, reflecting on the researcher’s starting points and relationship to the field becomes an important part of ethnographic work [67]. Such a ‘reflexive’ approach adds transparency to the analysis and contributes to validity. As described earlier, albeit a long time ago, I have worked in both psychiatry and the user movement, but I have no personal experience with clinical mental illness. One of the key preconceptions I brought to the study was that it is important not only to implement user involvement within mental health services but also that user perspectives are allowed to have a real impact in guiding services. In my view, if there is no such influence, then user involvement activities become merely tokenistic. Nevertheless, my ambition was to observe how user involvement was being implemented rather than to be an activist or an active part of that implementation. This passive role was largely accepted by the actors in the field, but as I was often present in the field, over time I was also given the opportunity to gather empirical material through informal discussions with members of the field before and after the formal activities I observed, as well as during breaks.

## 5. Results—The Market of Lived Experience

When the field of service user involvement is individualised, the emphasis on individual case influence and freedom of choice increases [59,69]. However, individualisation also affects user involvement at the organisational level [21]. Even though collective forms of influence were common in my study, the demand for personal testimonies—as opposed to the collective knowledge or viewpoints of the user group—also penetrated these forums. And in a context where user participation is increasingly influenced by market logic [22], a market for lived experiences of mental illness is taking shape. The analysis begins with an account of how the personal narrative is constituted as a commodity, followed by an exploration of how the market for these stories manifests. Finally, the extended market is explored, highlighting additional aspects of the commodification of users’ experiences.

## 6. Demand and Customisation—The Personal Narrative as a Commodity

Mental health user organisations have a long history of giving presentations on mental illness to public organisations, workplaces, and public events. The purpose has been to counter negative stereotypes or to draw attention to issues that the user movement is pursuing. Not least, public psychiatry has invited speakers from the user movement. However, the demand for the voice of the user movement is changing. Below is an extract from the PPO user council, where the management meets regularly with representatives of local user organisations. The discussion is about a staff training programme to be run jointly by the user movement and the PPO:

Viktor (user organisation representative): When we are out and about talking, we are giving our view of reality. But how will our view be expressed in such a joint project?

Kasper (PPO coordinator and mental health social worker): Well, I should clarify. The aim of this project is not to give the user organisations’ interpretation of the problems in society or in Swedish psychiatry...

Viktor: No, exactly.

Kasper: … it is just people expressing their personal experiences… The idea is to increase knowledge, to tell about their lives, to create contact, to talk to people. And of course everything can come up in these discussions. But it should not be a mouthpiece for the user movement.

Viktor: And it’s not a mouthpiece for the psychiatric organisation?

Kasper: No. What it is… it’s just the experiences of individuals.

(Transcription, User Council)

Experiential knowledge can take both individual and collective forms [70], but here the PPO is not asking for the aggregated experiences or collective perspective of the user organisations. Indeed, the PPO seeks to engage the user movement and involve its representatives, but to access the lived experiences of individuals; a practice that can be understood as an individualisation of the collective dimensions of user participation (see further [21]). This tendency was not unique to this staff training programme. In several forms of user involvement, patients and former patients were engaged to share their personal experiences, and usually the PPO paid them for their contribution. In this way, the personal narrative becomes a commodity that individuals can sell to the psychiatric organisation [15].

I have explored elsewhere how this commodity is structured [14] and how it is actively edited by the PPO through the selection and training of the individuals employed, as well as through the instruction of what the desired narrative looks like [71] (see also [15] p. 280). Consequently, it is not just any narrative that is sold, but a specific narrative that is adapted to the demand of the PPO. These narratives have a distinctly personal character and include experiences of both mental illness and the treatment the narrator has experienced. The narrative also follows a certain structure, in which expressions of dissatisfaction and criticism of the organisation are ‘embedded’ in constructive suggestions and examples of when the psychiatric organisation has done well (see [14] pp. 18–22). In addition, the ‘turning point’—i.e., when and why the individual’s health has improved and how mental health services have contributed to this—is a central aspect of the narrative that is required and enables the narrative to be sold within the PPO.

The PPO regularly organised training programmes to prepare patients and former patients to present their stories. During these sessions, participants were trained to speak in front of an audience and deal with different presentation situations. They also learned to adapt their story so that it could be received by the audience within the organisation, which largely meant not being too critical [14,15]. In addition, the training sessions contributed to the constitution of the personal narrative as a commodity. It could sound like this:

What you should do, says the tutor, is look at the purpose of your presentation and then think about your “product” in relation to that purpose. What is being asked for in this particular situation? Which parts of my story should I emphasise?

(Field note, Knowledge Dissemination Training)

Patients’ experiences are formulated as a ‘product’ that is in demand and adapted to the potential client/buyer. Before the training ended, one of the tutors talked about the participants’ future as ‘knowledge distributors’ within the PPO:

Assignments may not appear out of nowhere, says the trainer. You should not expect to suddenly get “a lot of work”. It depends on the demand for the type of mental illness you have experience of. Postnatal depression, for example, is probably a fairly “narrow niche”. Fatigue, on the other hand, is broader. But you don’t have to just sit and wait, you can actively “create your own job opportunities”! If you get together and actively look for work, you can create “events” where you “market” your services to potential clients.

(Field note, Knowledge Dissemination Training)

The act of telling one’s story is repeatedly referred to as ‘work’, and in this way, service users’ narratives are constructed as a commodity to be supplied by the individual. Different products (experiences) have different market prices, with fatigue probably being easier to sell than postnatal depression because the former is a bigger niche—a bigger market segment. Participants are also encouraged to actively ‘market’ their product. ‘It is all about marketing your knowledge,’ as one of the survivors who participated in the training programme concluded after the seminar.

The psychiatric user movement in Sweden and its umbrella organisation, the National Collaboration for Mental Health (NSPH), has adapted to (and probably helped to create) the demand for lived experience in Swedish mental health care, for example through a large-scale national project based on individuals sharing their stories of mental illness (see [72]). Given the persistence of negative attitudes towards people with mental illness [73], the narratives are expected to contribute to reduced stigma and improved mental health care [72]—logic supported by research [11]. In addition, personal narratives may have a rhetorical advantage over collective political narratives because their intimate nature can create an emotional connection between the listener and the narrator, generating a willingness to change [14]. Thus, the increased interest in personal experiences of mental illness and psychiatric treatment has the potential to achieve desirable effects, and as Razack [74] on p. 65 points out, it can be difficult to critically examine practices that have benevolent intentions, especially when they are carried out by individuals who are perceived as ‘good’ and themselves in a disadvantaged position. It is not my intention to criticise those who tell their stories or the fact that space is given to these stories. On the contrary, I argue that it is essential for mental health social workers to be informed by service users’ experiences [1]. Nevertheless, the commodification of personal experience needs to be problematised. What are the consequences of this commodification for the individual narrator and for the user movement’s ability to influence mental health services? The following critical reflections from individuals involved in the Canadian user movement highlight the potential dangers:

… In the last decade, personal stories have increasingly been used by the psychiatric system to bolster research, education, and fundraising interests./…/Personal stories from consumers/survivors have been harnessed by mental health organizations to further their interests and in doing so have shifted these narratives from ‘agents of change’ to one of ‘disability tourism’ or ‘patient porn’.

[13] p. 85

The authors emphasise that the narratives—their critical content, as well as their purpose and effects—are likely to change as they are incorporated into the mental health system. Jijan Voronka [15] argues that the commodified user narrative, in order to be marketed and sold, is forced into a form that may reproduce existing perceptions of mental illness and replicate existing organisational and social structures rather than contribute to social change and improved conditions (see also [12] p. 437, [13] p. 89, [14]).

### 6.1. Hopes of Employment and Ways of Selling the Personal Narrative

The demand for user narratives within the PPO, as well as in neighbouring human service organisations and society at large, led many service users to hope that they could enter this new (labour) market by selling their stories. Before a staff training session at the PPO, where psychiatric survivor Gunilla was asked to talk about her experience of case management (see [75]), we shared a coffee in the hospital cafeteria:

Gunilla says she is a bit stressed because in the next few days she will find out whether she can keep her job as a teacher at a primary school. It is a difficult situation, she says, because she really wants to keep working. But if she loses her job, she is thinking of starting to work by giving talks like the one she is giving today. She has heard from others that you can make a lot of money giving talks and writing books about your experiences as a psychiatric survivor.

(Field note, Staff Training)

Gunilla has become aware of the market that has been created for lived experience and perceives it as a potential alternative to her current employment. Many (especially long-term) psychiatric patients have an insecure position in the regular labour market and live in strained financial circumstances [10]. This opening of the door to a new labour market, where personal experience of mental illness is precisely what is in demand, can, therefore, be tempting because of the pressures of a social context in which employment is increasingly central to the perception of the individual as a full member of society [31,32]. The discussion resurfaced in the car on the way home from another staff training session, which two other user representatives had attended:

Sara (user representative) says that she thinks it’s great that they get paid by the PPO to speak, and the user representatives start to discuss what they usually charge when they give ‘private’ talks. 300 euros, says Sara. 600 euros, says Anders (user representative), who has more experience.

I didn’t know how much to charge, says Sara. So I emailed [a well-known author] and asked. She is a bit of an idol for me, and she has a lot of experience, and she charges 1000 euros. So I thought 300 would be OK.

Petra (PPO coordinator) says that if you are famous and have a few books in the back you can charge well, and Anders replies that it is probably not that easy for everyone.

Then they start talking about setting up their own business to do their lectures. Anders is already there, but Sara has not yet managed it. It was a mess with the social insurance: you cannot earn money as a self-employed person and be on sick pay at the same time.

(Field note, staff training)

There are several ways of disseminating (and selling) the personal story. Sara and Anders both *work voluntarily* in their respective user organisations and sometimes act as lecturers on behalf of these associations—a task that, due to the commodification of personal experience, is increasingly merging with and shifting towards *being hired* as a narrator on behalf of psychiatry. When the narrator volunteered to give ‘lectures’ on behalf of the user organisation, the personal experiences were usually supported by the recounting of the experiences of others and were often (and especially in cases where the user organisation was the organiser of the event) accompanied by more principled and critical statements that followed the official agenda of the user organisation [70]. In contrast, when the narrator was directly engaged by the PPO, the narratives tended to focus more exclusively on the narrator’s own experience, typically following what the narrator had been ‘asked to talk about’ by the PPO. Historically, I was told, the user organisations had been more active in organising their own talks and lecture series, but at the time of the research, it had become more common for such events to be organised in collaboration with the PPO or by the PPO alone—in a sense competing with the user organisations over human resources. As indicated in an earlier quote, several user organisations were concerned that this development would weaken their position and reduce their ability to convey their autonomous points of view. At the same time, several of the user organisations were suffering from declining numbers of members and volunteers, making it difficult to organise and manage large-scale campaigns on their own without the support of the PPO.

In addition to these two options, the alternative of *marketing one’s story ‘privately’* seems attractive, and people who have successfully marketed their experiences in this way are often held up as an ideal. Marketing one’s story privately can, but does not have to, mean that the individual runs a private (one-man) business. Acting privately, however, always means that the individual is marketing his or her story in an entrepreneurial way, perhaps also through publications and social media. This private practice illustrates that the market of lived experience is by no means exclusive to the PPO. The market for lived experience extends across society, where the same individuals who act within the PPO may also be contracted by other private or public organisations or market their experiences directly to the wider public. In summary, there are at least three ideal and typical positions as a narrator: (1) acting as a representative of the user movement, (2) acting as an employee and selling your work to a fixed organisation (in this case the PPO), or (3) acting as a private contractor marketing and selling a service/product to a wider market. In practice, these positions overlap and coincide, but the processes of commodification that are taking place mean that the first form is increasingly moving towards, or being trumped by, the latter two.

The extracts also highlight some of the difficulties associated with entering the market of lived experience. First, of course, not everyone can become a successful writer. Secondly, there is an inherent dilemma in the experience market because the experience you are selling requires you to be (or have been) ‘sick’, while operating smoothly in the market requires you not to be sick. This problem was raised by several of the service users in the study; the sporadic pay they received for their services made it difficult to receive their main source of income through health or social insurance.

### 6.2. Vacancies and Working Conditions

The PPO organised several activities in which patients were recurrently hired to share their experiences. But the PPO was not the only actor to appear as an organiser and employer in the market of lived experience. As already discussed, individuals could sell their experiences through their own businesses. In addition, other private market actors had taken an interest in this new market, offering similar ‘jobs’ as the PPO. The excerpt below is from a follow-up meeting of the semester’s ‘Enhanced Dialogue Initiative’—one of the PPO training programmes that focused on patient perspectives:

Petra (PPO coordinator) says that we can wait a few minutes before starting the meeting. Then she asks Carl (former patient) if Tina is coming (Tina is Carl’s girlfriend and also an educator within EDI). Carl replies that Tina cannot come because Anna Bergengren has “stolen” her. Carl tells us that Berggren is a trained social worker and runs a company that sells training in “recovery-oriented methods”. Carl says that Tina has become one of Bergengren’s “tell-your-story-girls” and that Tina is on an assignment with Berggren today. Carl goes on to say that Tina has also decided to skip a course she had signed up for at the university because she is already getting so much work through Berggren’s company.

Diana, an unassuming young woman sitting next to me, leans over and whispers that she is curious about how much you can earn as a narrator because she has also been thinking about starting her own business.

(Field note, follow-up meeting)

The excerpt shows how other market actors—in this case, former mental health social worker Anna Bergengren, who runs a business selling training in leadership, recovery, empowerment, case management, and other related areas—have perceived the demand for the lived experience of mental illness and consequently started to hire people with such experiences to tell their stories. One way of interpreting this kind of public storytelling is as a strengthening of the position of mental health service users because they are being listened to. At the same time, the mutual positions of the actors need to be considered. If so, the situation can also be understood as a non-reciprocal communicative relationship [76]. In this situation, a large audience consumes the storyteller’s most intimate (and by the organiser’s controlled and edited) narratives without giving anything in return to the narrator. (Several scholars have interpreted this as an expression of power asymmetry, where individuals from certain groups have to share private or intimate experiences in order to be listened to [13,16,77].) When companies like Bergengren’s hire individuals to narrate their personal experiences, the audience instead pays the organiser—in this case Bergengren’s company—to listen. In Marxist terms, the narrators sell their labour while the added value of their work generates an economic profit for someone else.

Such added value is not always of a monetary nature. For example, the storytelling that takes place within the PPO does not generate (direct) monetary gains. However, it does generate other added values for the organisation. Most obviously, users’ stories contribute experiential knowledge that can be utilised within the organisation [78]. In addition, both Costa and colleagues [13] and Voronka [15] show that users’ storytelling generates added value that goes beyond the actual content of the narrative. According to the authors, the mere fact that users are invited to speak creates legitimacy for the psychiatric organisation. Furthermore, the psychiatric organisation may also use users’ stories to pursue and support its own agendas and processes, which may not necessarily be in line with users’ intentions or interests (ibid).

For Tina in the extract above, the prospect of selling her experience has apparently become lucrative enough to compel her to give up her place in higher education. So for some people, at least in the short term, this market appears to be a viable way of making a living. But to what extent can you make a living from telling your story? For how many people is this a real possibility? How favourable are the working conditions? Based on my observations, it seems that the market of lived experience is characterised by temporary, uncertain, and precarious [32] working conditions. Permanent employment was rare. Instead, storytellers typically received an hourly fee for each individual job. Furthermore, as Voronka [15] points out, by no means all personal narratives are in demand. Nevertheless, within the PPO, patients are encouraged to orient themselves towards this (labour) market and many see it as an opportunity to earn money from the particular illness that has made it difficult for them to engage in the regular labour market. For some (especially high-functioning individuals already established in the regular labour market), the opportunity to market their story can become an exciting side-line. For others, it may be more difficult to enter the market of lived experience. Speaking in front of an audience, writing autobiographical books, running a business, and ‘creating your own job opportunities’ requires creativity, entrepreneurial spirit, and perseverance (as well as material conditions) that may not be available to all. Some years later, when I searched the web for any storytelling businesses started by individuals in my study who—like Gunilla, Sara, and Diana in the extracts above—expressed a willingness to pursue such endeavours, I did not get any hits, suggesting that not everyone’s hopes were realised.

Another important aspect to consider is how those who operate in the market of lived experience perceive the situation and what it means that the commodity they are selling to is based on their mental illness. Sharing one’s story is not necessarily an undivided positive experience; it can also imply vulnerability. As Jijan Voronka writes:

In this dynamic of being complicit in sharing your narrative, often out of financial need, yet not being in control of what angles are highlighted by the audience, unfamiliar with who views it, how it is consumed, and cognizant that others are reaping benefits far larger than you through the process… I have felt pleasure and shame, guilt and joy when the applause follows.

[15] p. 272

Based on interviews with people who work with sharing their experiences, Voronka [15] p. 278, p. 290 highlights how they have experienced a sense of loss of control and reproduction of stigma and dependency. Furthermore, if an individual’s mental illness is crucial to their work, there is a risk that they will cultivate an identity as mentally ill, a role that they would otherwise benefit from moving beyond [16]. This is especially true for those who give up other opportunities for employment or education in order to operate in the market of lived experience.

## 7. The Extended Market

So far, the article has focused only on the commodification of personal stories, but the commodification of service users’ lived experiences also took other forms in this study. In the following section, I will look at two examples of this ‘extended market’.

### 7.1. Personal Experience as a Qualification for Employment

Petra was one of the people employed full-time by the PPO to implement user involvement practices and was recruited partly because of her personal experience with mental illness. She left her job as a manager in a youth centre to take on this role. Thus, the implementation of user involvement within the PPO created job opportunities in which experience of mental ill-health became an asset for appointment. This is reflected in the increasing demand for ‘experts by experience’ in various areas of the social care sector (e.g., [20]). Indeed, the experience of mental illness was also a sought-after qualification in other recruitment processes within the PPO. With so-called ‘peer (support) workers’ [17,79] as role models, there was a growing interest within the PPO to recruit treatment staff with open personal experiences of mental ill-health. This is the formulation of a job advertisement as a ‘user expert’ within PPO substance use treatment:

We are now looking for two user experts to be based at the MARO clinic (Medical Assisted Rehabilitation of Opiate Dependence). As a user expert, you will hold individual meetings with users at the start of treatment, as well as host family programmes. You will also design and deliver patient education and produce information material. In your specific role, you will provide support based on your own and others’ experiences of recovery. You will provide professional peer support, work to build hope and confidence in participants and provide strategies for coping with illness.

To apply, you must have personal experience of treatment at a MARO reception. You are keen to share your knowledge and experience with other team members and develop your own.

(job advertisement)

A core idea of employing people with personal experience is to provide ‘user-centred’ services within the organisation. In addition, these employees are expected to have a special understanding of the recovery process and thus be able to support patients in a way that other professionals presumably cannot [79]. This is another way of commodifying lived experience. It is no longer the personal story that is being marketed. Instead, one’s personal experience becomes a merit for employment as a staff member within the PPO. In this case, the individual enters into a regular employment relationship with the PPO, which can be seen as a far-reaching commodification of lived experience. In some forms of substance use treatment, personal experience has long been a requirement for appointment as a staff member, but similar ideas are now spreading to wider areas of mental health and social services [79]. In Assertive Community Treatment (ACT), for example, it is a strong part of the methodology that at least one person on the team should have experience with problems like those being treated, and the PPO’s ACT team did indeed employ ‘user experts’.

### 7.2. The User Movement as Business Developer

The tendencies towards commodification appear at two different levels: the individual level—which has been addressed so far in the text—and the collective level. At the collective level, commodification is expressed in the fact that user organisations are beginning to act more on the basis of market logic in their relationship with the public. As the title of Alm Andreassen’s article [28]—*From Democratic Consultation to User-employment*—suggests, the phenomenon of user involvement is shifting from a democratic discourse based on the idea of power-sharing and joint decision-making to a discourse of ‘co-production’, which focuses on the joint provision of high-quality services. This shift is well-reflected in the way user involvement was formulated within the PPO. Although democratic discourse was sometimes still strong—especially in activities such as user councils and other dialogue-oriented activities—at the same time, activities were often framed in terms of ‘joint organisational development’, illustrating how different logics of user involvement coexist in practice (see [40]). Different logics are not always mutually exclusive, but how an activity is formulated affects how actors understand and act in the situation [80]. For example, the same activity (e.g., user representatives participating in a working group within the PPO) can be formulated primarily in democratic terms or primarily in terms of co-production. (Like the concept of ‘user involvement’, ‘co-production’ is itself a diverse and multifaceted concept, subject to different etymologies and interpretations, but in its contemporary application it can be argued that this concept moves closer to market logic than democratic logic [28]. However, whether participatory practices are called user involvement, co-production, or something else, what they produce in terms of democratisation, empowerment, and improved services is ultimately an empirical question that depends heavily on how the concepts are implemented in practice [40]. But, as argued here, a shift in terminology from user involvement to co-production—with its semantic connotations of market terminology—is unlikely to help mitigate the tendencies to commodify personal experiences of mental illness.) Many involvement activities within the PPO were predominantly formulated in terms of co-production, which had implications for how the parties related to each other. This reveals another aspect of the commodification of service users’ experiences, highlighting how user involvement is increasingly influenced by market logic [22]. In addition to acting as an independent voice and safeguarding the interests of service users—a description that the user movement still holds dear—the collective user movement is increasingly perceived (and perceives itself) as an external party, hired to support the organisational development of public organisations. Here, the representatives of the user movement become ‘consultants’ whose ‘services’ are purchased by the public. Examples of such services purchased by the PPO included user-led audits, organising self-help groups, providing user-expert guidance to staff groups, and conducting staff training (see [40]). Non-profit user organisations delivering social services through procurement are another example of this development [62]. This commodification of user involvement at the collective level implies that the democratic ambitions that previously characterised these activities may lose ground [21,28]. User movement is no longer just an advocacy organisation and increasingly resembles a consultancy or recruitment agency for personal experience—and sometimes also a competitor among other providers in the welfare market.

## 8. Discussion—Changing Conditions for Influence

The analysis has shown how user involvement initiatives in contemporary mental health care tend to commodify users’ personal experiences, and I will now discuss some potential consequences of this development. The tendency towards commodification has implications for how service user representatives operate when attempting to influence the welfare agency by creating specific conditions for action. The commodification of the personal narrative affects the way in which user representatives communicate their experiences, and in the extended market, both individual service users and the user movement increasingly begin to act as employees or contracted business developers in relation to the PPO. As mental health social workers often take on the role of implementing user involvement within mental health services, it is crucial for them to be aware of this development and its potential consequences.

### 8.1. Towards an Employment Relationship

As user involvement practices become increasingly influenced by market logic, the idea that user representatives should be paid for their participation is emphasised and normalised. From the perspective of the user movement, remuneration is advocated as a matter of equality, legitimacy, and power (see [81]). Sometimes the issue is also expressed more directly: ‘If we are involved in the “quality work” of public mental health services, why shouldn’t we be paid’, as one user organisation representative put it. Market logic makes such formulations possible. In a situation where user involvement is formulated less in terms of democratic influence and more in terms of carrying out a task on behalf of the organisation—as ‘educators’, ‘peer workers’, ‘counsellors’, or ‘supervisors’; roles reminiscent of professional work—it becomes natural for user representatives to be paid.

The PPO regularly offered compensation (hourly rate and travel allowance) to user representatives who participated in user involvement activities. In addition to potentially empowering the user representatives, this payment enabled the participation of individuals who were unable or unwilling to participate on a voluntary basis. In addition, the payment was seen as creating a more ‘proper’ participation of user representatives. Representatives from both the user movement and the PPO expressed that the payment made it possible to ‘make demands’ on the user representatives to carry out their duties in a ‘serious way’ [40] p. 137. Similar rhetoric suggests that the remuneration has the potential to allow the PPO to influence the behaviour of the user representatives involved. As expressed in the study, the remunerated user representatives are expected to ‘act professionally’ (note the connotation with market terms) and ‘not be obstructive’. This suggests that remuneration may increase the likelihood that the service user representatives will comply with, rather than contradict, the overarching intentions of the PPO.

In part, the practice of user involvement shifts from being a democratic action to a job that an individual or association is paid to do. This may have more profound consequences than is generally recognised because it changes the relationship between the parties. In neoliberal terms, service users are constructed as involved customers [18], but through remuneration, participating service users approach the position of an employee, creating confusion between the positions of citizen, consumer, and producer of welfare [82]. Etzioni [83] emphasises financial compensation as the main reason for employees’ compliance with the management of an organisation; employees are expected to be loyal to the employer. Although the compensation of user representatives cannot (usually) be equated with regular employment, the compensation creates a similar bond between the user representatives and the PPO. These bonds weaken the user representatives’ (and user organisations’) position as an independent party and their ability to act on the basis of an independent agenda [21]. Essentially, they adapt to the job description prescribed by the buyer of their labour and carry out the kind of engagement that the organisation asks for.

The remuneration of user representatives can be seen as a double-edged sword. It can provide a much-needed economic contribution to the individual, enable the implementation of user involvement practices, legitimise peer workers as skilled actors within social service organisations, and possibly also provide recognition to user representatives and the user movement. At the same time, remuneration creates new types of bonds between actors that risk suppressing criticism and counteracting more radical change [15,21]. At the same time, there is a risk that the link between user involvement practices and democratic logic will be weakened because the economic remuneration offered by public human service organisations may create incentives for individual patients to reduce their voluntary involvement in the collective user movement and instead sell their labour (or services) directly to public human service organisations [21].

### 8.2. The Individual and Identity

According to Marx, the commodification of labour meant that individuals were deprived of part of their identity or humanity. However, Marx’s theory of alienation has been criticised for assuming a basic human nature that is distorted or lost when labour is commodified and organised in a market (see, for example, [84] p. 224). In a society where work/occupation/employment has become key to the very definition of individual identity [31,32], it can be difficult to imagine a different order. Nevertheless, it is important to reflect on the impact of commodification on the individual, especially in this case where it is specifically lived experiences and individual life stories that are being commodified. In the market of lived experience, only certain narratives, experiences, and identities are in demand. Narratives and personalities that do not fit are not acquired—something that individuals must adapt to in order to operate in the market [14], [15] p. 281, [71]. Thus, it could be argued that the formation of a market of lived experience de facto shapes the identity of individuals by encouraging people to edit and change the way they express their narratives and experiences. Voronka [15] highlights the loss of control over one’s life story as a problematic aspect of the commodification of personal experience, which could indeed be interpreted as an expression of alienation from the self.

### 8.3. Implications for Mental Health Social Workers

Finally, let us take a step back and consider the implications of the analysis for social workers in general and mental health social workers in particular. For example, it may mean that we get more colleagues with open experiences of mental illness and that stigmas about sharing and using such personal experiences within the work group may be reduced (see [85]). It may also lead to recognition and appreciation of experiential knowledge within mental health services. At the same time, caution is needed.

In the empirical extracts from the analytical section above, mental health social workers occasionally appear as representatives of the PPO. Indeed, in the study as a whole, mental health social workers (followed by nurses and care assistants) were the profession most actively involved in implementing user involvement activities, while psychologists and psychiatrists were only occasionally involved. This is perhaps unsurprising given that social work training emphasises the importance of recovery-based perspectives, participatory practices, and experiential knowledge [17,86,87]. It is, therefore, important, particularly for mental health social workers, to remain vigilant and critically aware of what kind of practice we are creating and promoting when we implement user involvement. There is no doubt that the mental health social workers and other professionals I met in the PPO—Kasper, Petra, and many others—were committed and well-meaning professionals who worked hard to create the best possible circumstances for service users. However, embedded in a contemporary market society, it can be difficult to recognise (and challenge) the processes of commodification of lived experience that takes place in our own practice, as it merges with broader societal processes and market logic that shape our way of thinking and understanding the world. As mental health social workers are active subjects in the local formulation and implementation of user involvement, we are also inevitably—consciously or unconsciously—agents of commodification processes. Indeed, mental health social workers should welcome and promote increased attention to service users’ perspectives and lived experiences within mental health services [1]. At the same time, we need to actively work to limit the potential negative consequences of commodifying lived experience in the implementation of user involvement.

Following my analysis, this means staying critically aware and reflexive about one’s own practice [17]. It means actively working to create an open climate that allows for a diversity of voices and identities, rather than narrowing the possible subject positions of user representatives [88]. It means being responsive to service user interests and supporting alternative perspectives—for example, by allowing service user representatives to formulate activities and set meeting agendas—rather than dictating and perpetuating the dominant practices and paradigms of the mental health agency [89,90]. It means seeking to safeguard the democratic logic that underpins the tradition of user involvement by working with activities that include collective voices and promote collective influence, rather than giving in to the trend towards streamlined individual storytelling. It means actively seeking to enable service user representatives and user organisations to maintain an autonomous position and to support service users in pursuing the issues that are important to them [91]. Not least, it means making conscious and considered—rather than routine—decisions about when, why, and in what form service user representatives are paid for their participation, and seriously assessing and managing the potential downsides of the practice of payment. For example, when the public welfare system engages service users in participatory activities, instead of paying individual participants directly—which risks disconnecting the individual service user from the user collective and its support structure—funding for representation could be managed through service user organisations so that the individual representative is formally paid by the user organisation rather than the welfare organisation. This would reduce the risk of losing collective perspectives and the erosion of user movement. Other ways of dealing with the disadvantages of payment are to support user organisations to become economically independent—which would reduce their need to adapt to the expectations of welfare organisations in order to secure funding—or limit the detailed management of paid user representatives, allowing them to work freely and pursue the service users’ agenda.

Critical awareness and insight into the processes of individualisation and commodification that are taking place, followed by such reflexive practice performed by mental health social workers implementing user involvement, can hopefully prevent the most negative effects of the commodification of lived experience and maintain a vital, inclusive, and meaningful user involvement. Individual user narratives can illuminate practice and provide important insights into how practice should be conducted, especially if they are expressed freely by service users and not edited by the service provider. But to avoid the negative effects of commodification when implementing user involvement—or co-production as it is now more commonly termed—it is important to recognise how these individual narratives contribute to the construction of a larger collective narrative of the user group [70]. Only from the aggregated collective experiences of the service user group can a common agenda for democratic action be formulated. Therefore, in order to keep the democratic aspects of user participation alive, it is important to continue to work with activities where user representatives are enabled to articulate their collective voice and argue on behalf of the user collective, such as user councils and user representation in decision-making bodies. In essence, this means that—in the mix and combination of different roles that service users are assigned in contemporary commodified user involvement practice, such as narrators, consultants, co-producers, evaluators, peer workers, etc.—it is important to enable service user representatives to retain their role as democratic actors.

## 9. Conclusions

The analysis has shown how the practice of user involvement, conducted in an era where society and welfare are characterised by individualisation [29], market logic [92], and commodification [30], increasingly takes the form of a market where personal user stories and experiences are bought and sold. One consequence of this is that the collective dimensions of user participation are being individualised through an increasing focus on individual narratives. Another potential consequence is that the role of user organisations as independent actors working for democratic influence has shifted to a role in which business developers work on behalf of public human service organisations based on the institutional logic of these organisations [13,15,21]. For those individuals engaged in user involvement activities, commodification means more opportunities to be compensated for their efforts, and their engagement is also transformed into something similar to a job, where the individual’s ability to communicate their personal experiences in an attractive way becomes an increasingly important prerequisite for being able to sell their labour or services in competition with others in the market of lived experience.

The ability of service users to effect change within a human service organisation is often dependent on the views expressed not being too far outside the organisational logic, i.e., being perceived by organisational representatives as ‘possible’, ‘realistic’, or ‘desirable’ to implement [40,93,94]. As the processes of commodification highlighted in the article seems to result in user representatives adapting their perspective to the organisational logic, this development could actually contribute to increased opportunities for influence. At the same time, the analysis shows that commodification also contributes to the suppression of more critical voices, which risks limiting opportunities for influence, as certain views may never be expressed.

## Data Availability

The anonymised data presented in this study are available upon request from the corresponding author. These data are not publicly available due to restrictions of confidentiality.
